# Dynamics of HIV Latency and Reactivation in a Primary CD4+ T Cell Model

**DOI:** 10.1371/journal.ppat.1004156

**Published:** 2014-05-29

**Authors:** Pejman Mohammadi, Julia di Iulio, Miguel Muñoz, Raquel Martinez, István Bartha, Matthias Cavassini, Christian Thorball, Jacques Fellay, Niko Beerenwinkel, Angela Ciuffi, Amalio Telenti

**Affiliations:** 1 Department of Biosystems Science and Engineering, ETH Zurich, Basel, Switzerland; 2 Swiss Institute of Bioinformatics, Basel and Lausanne, Switzerland; 3 Institute of Microbiology, Centre Hospitalier Universitaire Vaudois, Lausanne, Switzerland; 4 School of Life Sciences, École Polytechnique Fédérale de Lausanne, Lausanne, Switzerland; 5 Service of Infectious Diseases, Centre Hospitalier Universitaire Vaudois, Lausanne, Switzerland; 6 University of Lausanne, Lausanne, Switzerland; Fred Hutchinson Cancer Research Center, United States of America

## Abstract

HIV latency is a major obstacle to curing infection. Current strategies to eradicate HIV aim at increasing transcription of the latent provirus. In the present study we observed that latently infected CD4+ T cells from HIV-infected individuals failed to produce viral particles upon *ex vivo* exposure to SAHA (vorinostat), despite effective inhibition of histone deacetylases. To identify steps that were not susceptible to the action of SAHA or other latency reverting agents, we used a primary CD4+ T cell model, joint host and viral RNA sequencing, and a viral-encoded reporter. This model served to investigate the characteristics of latently infected cells, the dynamics of HIV latency, and the process of reactivation induced by various stimuli. During latency, we observed persistence of viral transcripts but only limited viral translation. Similarly, the reactivating agents SAHA and disulfiram successfully increased viral transcription, but failed to effectively enhance viral translation, mirroring the *ex vivo* data. This study highlights the importance of post-transcriptional blocks as one mechanism leading to HIV latency that needs to be relieved in order to purge the viral reservoir.

## Author Summary

HIV-infected individuals must receive lifelong antiviral therapy because treatment discontinuation generally results in rapid viral rebound. The field has identified a state of latency at the level of transcription of the integrated provirus as the major mechanism of persistence. A number of drugs are now tested that aim at inducing viral transcription as a step to purge the reservoir. The assessment of viral production in cells from HIV-infected individuals with optimal viral suppression revealed the failure of SAHA/vorinostat to efficiently generate viral particle production. To further investigate and characterize the process of latency at the transcriptome level, and the response to SAHA as well as various reactivating agents, we use a model of primary CD4+ lymphocytes. The main observation from this study is that viral transcripts persist during latency, and that the accumulation of viral transcripts does not result in efficient viral protein expression upon reactivation with agents such as SAHA. Our data suggest that post-transcriptional blocks also contribute to latency, and that additional strategies need to be explored to efficiently purge the viral reservoir.

## Introduction

Successful antiretroviral therapy reduces HIV plasma viremia to undetectable levels; however, the virus is not eradicated [1]. Current and non-mutually exclusive hypotheses to explain this phenomenon include (i) the presence of anatomic viral reservoirs that are inaccessible to drugs, (ii) ongoing viral replication and cell-to-cell spread, and (iii) the existence of a long-lived cellular reservoir that is infected but does not produce viral particles and can be reactivated. With the last hypothesis, it is proposed that reactivating viral transcription in latently infected cells may help purging the HIV reservoir and contribute to HIV eradication [2]. Therefore, a better understanding of the mechanisms involved in viral transcriptional silencing and in the reactivation from latency is essential. Less attention has been paid to post-transcriptional blocks which prevent completion of the viral replication cycle, thus limiting expression of viral proteins, *de novo* production of viral particles and cytopathogenesis.

Currently, there are two competing models on how HIV establishes latency in resting CD4+ T cells [3], [4]. The first model suggests that activated cells are infected by HIV, and that most of them are productively infected and die within a few days. However, a minority of cells revert to a resting memory state, following the natural biology of CD4+ T cells. The second model proposes that HIV is able to directly infect resting CD4+ T cells, even if this process is poorly efficient. To study HIV latency experimentally, cell lines have been used extensively to investigate the molecular mechanisms underlying impairment of viral gene expression. Cell lines highlighted viral transcriptional silencing as a major determinant of HIV latency (reviewed in [5]–[8]). There is however increasing interest for work in more representative primary cell settings. In recent years, multiple systems using primary cells from various CD4+ T cell subpopulations have been developed. These systems yield sufficient numbers of cells allowing investigations that may recapitulate more closely *in vivo* processes (reviewed in [4], [9].

Primary cell latency models have been used for the screening and assessment of molecules that promote viral transcription [10]. Multiple compounds [11] reactivate viral transcription from latently infected cells *in vitro*, reflecting the various mechanisms involved in viral transcriptional control, including epigenetic regulation (such as histone modifications and DNA methylation) and immune modulation (such as T cell receptor engagement and protein kinase C signaling) [8], [11], [12]. The efficiency of these agents varies according to the HIV latency model used [11], [13], underscoring that the mechanisms leading to repression and reactivation of viral transcription may be cell-specific. Some of the agents are moving forward to clinical trials [1], [11], [14]–[16].

We have previously completed a detailed analysis of the cellular response to HIV infection in a T cell line and derived a model of cell reprogramming upon viral invasion [17]. Here, we use an extension of this experimental approach to investigate viral latency and reactivation in a model of primary CD4+ T cells. We hypothesized that a comprehensive assessment of the paired transcriptomes of the host and the virus by RNA sequencing, coupled to the analysis of viral protein expression, would allow for addressing the extent of viral transcriptional and post-transcriptional silencing, the structure of the viral transcriptome, and the nature of the cellular transcriptional program induced by pharmacological and immunological reactivating compounds. Our *in vitro* and *ex vivo* data reveal defects in viral translation and particle production that reflect a state of post-transcriptional cellular latency which may not be reversed by agents merely enhancing viral transcription.

## Results

### Viral production from latently infected cells *ex vivo*

**Figure 1:**
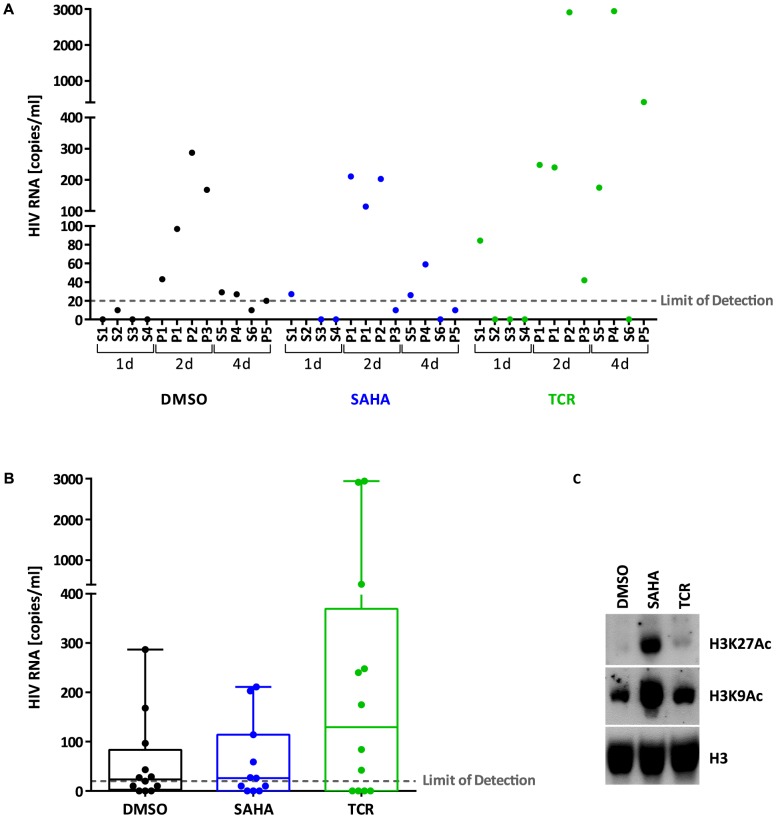
Viral production from latently infected cells *ex vivo*. Panel A. HIV RNA copies/ml measured in the supernatant of resting CD4+ T cells isolated from treated HIV+ individuals (S: cells from single individuals, P: pooled cells from 2-3 individuals) and stimulated *ex vivo* with DMSO (control, black), SAHA (blue) or TCR (green) for 1, 2 or 4 days. Panel B. Box plot showing HIV RNA copies/ml measured in the supernatant of resting CD4+ T cells isolated from treated HIV+ individuals and stimulated *ex vivo* with DMSO (control, black), SAHA (blue) or TCR (green) for 1, 2 or 4 days. Each circle represents a different cellular sample. Bars: min to max. Solid line: median. Panel C. Immunoblot of histones (1 µg) extracted from stimulated *ex vivo* cells isolated from treated HIV+ individuals. H3K27Ac: anti-histone 3, acetylated Lysine 27; H3K9Ac: anti-histone 3, acetylated Lysine 9; H3: anti-histone 3 (total).

We compared the efficacy of T-cell receptor (TCR) stimulation and SAHA treatment to stimulate viral particle production *ex vivo*. For this, we tested virus release from latently infected cells isolated from HIV-infected individuals under successful treatment (**Table S1**). We isolated resting CD4+ T cells and cultured them for 1, 2, and 4 days in the presence of IL-2 supplemented with DMSO (control), SAHA or TCR stimulation. TCR stimulation resulted in successful viral particle production in 7 of 11 samples, with viral RNA copies ranging from 42 to 2940 copies/ml of supernatant. Viral RNA copies in controls (mock-treated cells) were at or below the limit of detection of the assay for 6 samples, and values ranging from 27 to 287 copies/ml for 5 samples (**Fig. 1**). SAHA was undistinguishable from control, suggesting a failure to stimulate particle production from latently infected cells *ex vivo*, confirming previous studies [18]. We observed effective inhibition of histone deacetylases confirming that primary cells were exposed to appropriate concentrations of SAHA (**Fig. 1**).

### Stability of the cellular model of latency

**Figure 2:**
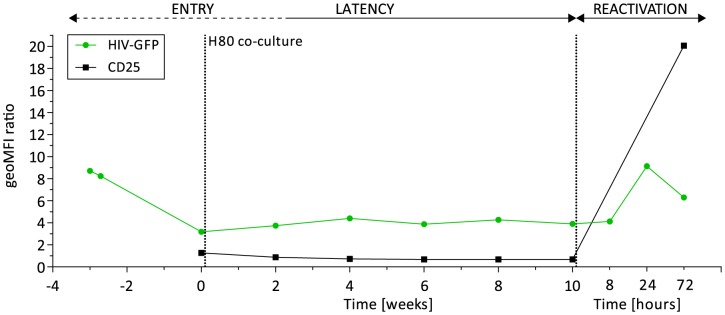
Dynamics of HIV latency in a primary CD4+ T cell model. Viral-encoded GFP expression (green) and activation marker IL2Rα (CD25) expression (black) during the steps of entry into latency (week -3 to week 2), maintenance of latency (week 4 to 10), and reactivation upon TCR stimulation (8, 24, and 72 hours after week 10). Shown are the geometric mean fluorescence of intensity (geoMFI) ratios of HIV-based vector infected cells and mock non-infected control cells.

To further investigate the mechanisms underlying SAHA inefficiency to reactivate latently infected CD4+ T cells *ex vivo* and generate viral particles, we used the cellular model described by the laboratories of Cloyd [19] and Karn [20] that preferentially produces latently infected CD4+ T cells with a central memory phenotype. These cells constitute the major cellular reservoir of HIV in blood [21]. The viral vector, previously used in latency studies, encodes Tat, Rev, and a destabilized GFP protein with an estimated half-life of 6 hours [22]. Starting with the transduction of two million cells from a healthy donor, we sorted a population of 200,000 successfully infected cells and amplified them to obtain 50 million cells. The infected cell population was then co-cultured on a feeder layer of the H80 human brain tumor cell line over 10 weeks (**Fig. S1**). We obtained a population of latently infected cells carrying a stable number of viral integration events, measured at 1.06 proviruses per cell. This measure of quality control, as well as the quasi-universal reactivation of the cell population upon T cell receptor (TCR) stimulation with anti-CD3/anti-CD28 and IL-2 (see below) excluded outgrowth by non-infected cells. The expression from the *env* open reading frame of a destabilized viral-encoded GFP decreased significantly following infection (*entry phase*), and reached its nadir upon co-culture on feeder cells (week 0). However, low level GFP expression remained stable at around 3-fold above background (mock uninfected cells) through 10 weeks of co-culture (*latency phase*) (**Fig. S2**). Upon reactivation by TCR stimulation (*reactivation phase*), we observed up to 9-fold increase of GFP expression; with 92% of cells expressing the viral-encoded GFP, **Figure 2**. The activation marker IL2Rα (CD25) remained undetectable during the latency phase. Thus, the model recapitulates the process of latency, although the basal degree of GFP expression suggests incomplete silencing of HIV.

### Incomplete transcriptional silencing of HIV

**Figure 3:**
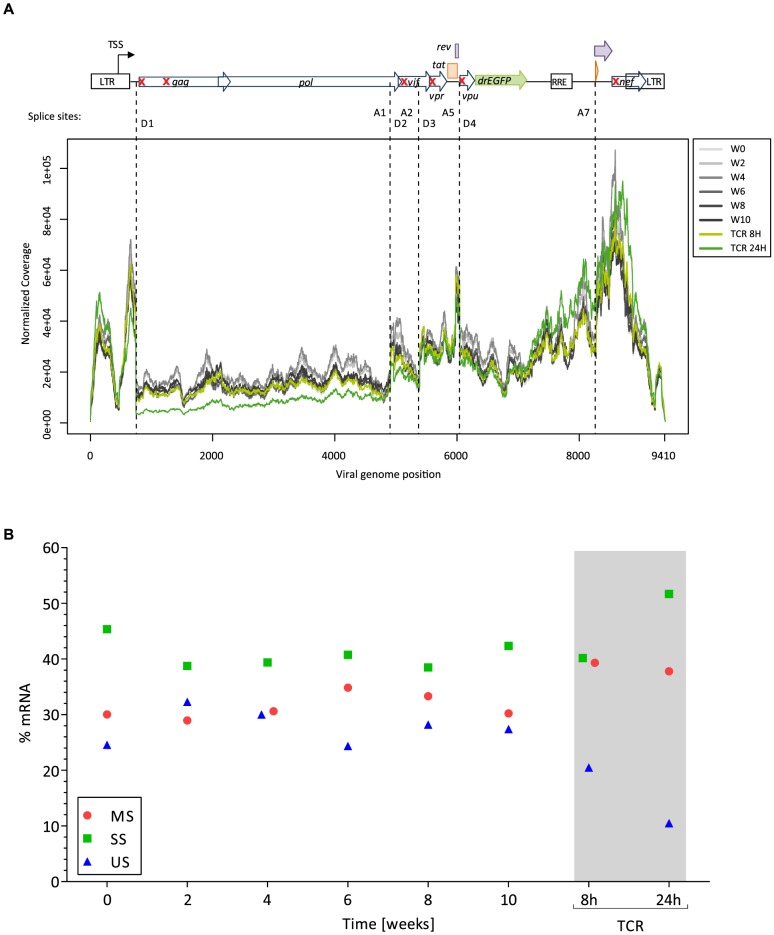
Features of HIV transcription. Panel A. Distribution of HIV reads along the vector genome. On the top is depicted the viral vector genome used (NL4-3-Δ6-drEGFP). Red crosses indicate the genes that are disrupted by stop codon insertion, frameshift or deletion. TSS: transcription start site; D: splice donor; A: splice acceptor. Reads mapping to the LTR are equally assigned to 5′ and 3′ ends, explaining the presence of viral reads upstream the TSS. Panel B. Pattern of splicing for the main viral RNA forms: genomic unspliced full-length viral RNA (US, blue), singly spliced RNAs without the Gag-Pol major intron (SS, green; spliced in D1 but not in D4), and multiply spliced subgenomic mRNAs (MS, red; spliced in D1 and in D4).

To understand the extent of viral silencing during latency, we assessed three parameters of viral transcriptional activity using RNA sequencing data. First, we estimated the proportion of viral transcripts among the total number of detected transcripts. From week 0 (W0) through week 10 (W10), this proportion was stable with a mean of 1.23% (95%CI; 1.15, 1.32). Second, we evaluated the distribution of viral reads along the viral vector genome. We identified a conserved pattern of coverage of the viral vector genome through the latency phase (**Fig. 3A**). Third, we assessed the pattern of viral splicing. All main splice variants were well represented and in conserved proportions [23] (**Fig. 3B** **, Fig. S3, Fig. S4**). Upon TCR stimulation, there was a proportional shift in viral splice forms. Indeed, cell activation resulted in a profound modification of the transcriptome and doubling of the RNA content of the blasts.

The RNA sequencing approach captures polyadenylated viral RNA (*i.e*. fully elongated). On these RNA species we did not observe major deficits in viral genome coverage, or splicing during latency. Thus, these viral transcripts could contribute to the residual viral-encoded GFP expression. However, these analyses do not discriminate between continuous translation from a stable pool of viral transcripts from the original infection and *de novo* transcription from leakiness of the latency model.

### Dynamics of entry, latency, and reactivation

**Figure 4:**
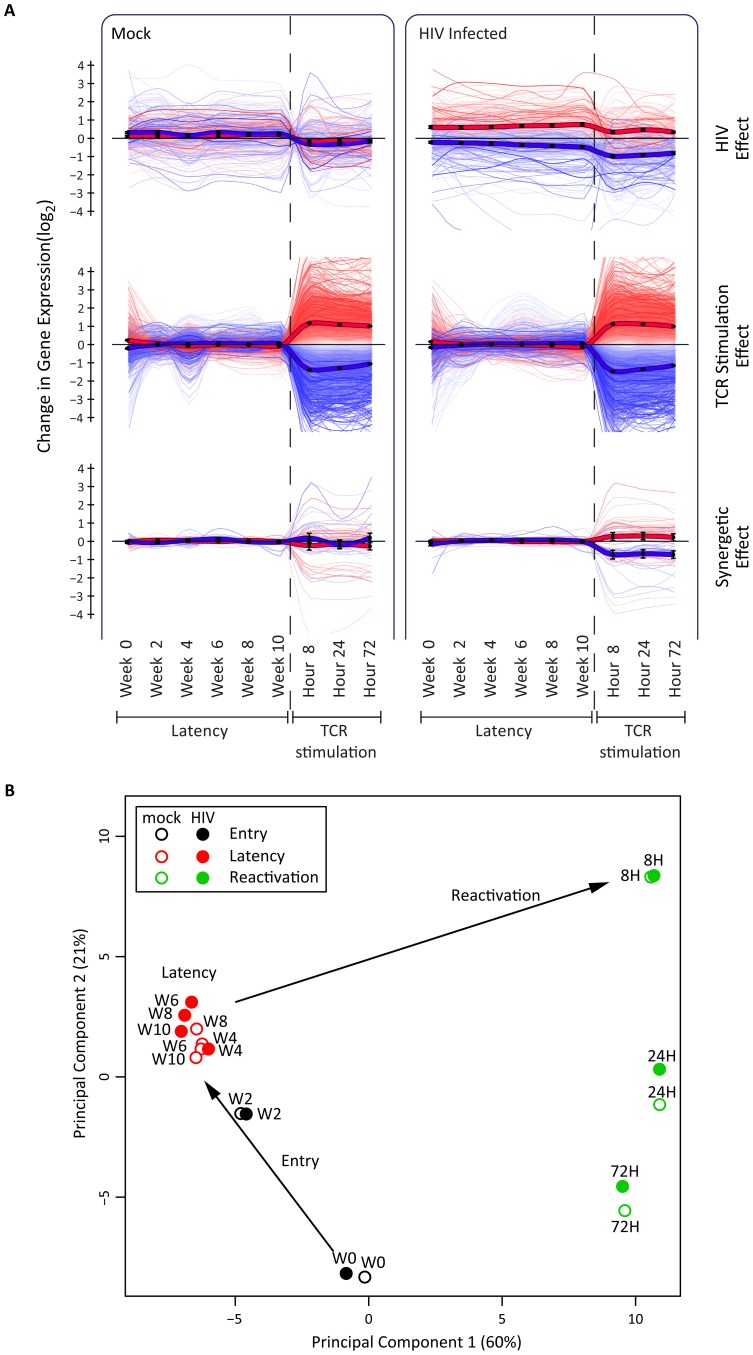
Host transcriptional response in latency and reactivation in a primary CD4+ T cell model. Panel A. Illustrated are 9729 genes with statistically significant association to at least one of the two experimental conditions, HIV infection and TCR stimulation, as evaluated by 2-way analysis of variance of 14513 expressed genes (FDR<0.05). Data are presented in three non-exclusive groups: those regulated in concordance with viral presence (345 genes, top panel), those regulated in concordance with TCR-stimulation 9647 genes, (middle panel), and those showing non-additive synergetic effects by viral presence and TCR stimulation (59 genes, bottom panel). The bold lines represent the group average, and color intensities are proportional to statistical significance of the effects within each group. Panel B. Principal component analysis of host whole-transcriptome data supports a three-step model of entry-latency-reactivation. There is a transcriptionally coherent latency phase between week 4 and week 10, and a distinct reactivation phase at 8, 24, and 72 hours after TCR stimulation. All data, including differentially expressed genes and enrichment analysis, are available at http://litchi.labtelenti.org.

The host RNA profile during the dynamic process of latency was studied in time-course transcriptome analyses by grouping host genes according to their transcriptional response to HIV and to TCR stimulation with anti-CD3/CD28. Whereas the virus only minimally perturbed the transcriptional state of the cell, TCR stimulation resulted in massive cell reprogramming, impacting 66% of all detected genes. Numbers of genes differentially expressed under the various conditions are detailed in the legend of **Figure 4**.

Principal component analysis revealed two transcriptionally distinct phases: entry (W0–W2) and latency (W4–W10) (**Fig. 4B** **, Fig. S5**). The tight cluster of samples during the 6 weeks of latency includes both infected and uninfected samples, indicating a stable process that is only minimally perturbed by the virus. To assess the differences between infected and uninfected cells, we compared gene expression in paired samples to pinpoint potential markers of HIV latency, consistently differentially expressed during the 6 weeks of maintenance of latency (W4–W10): we identified 103 cellular genes as upregulated and 124 as downregulated in HIV-infected samples during this phase. The differentially expressed genes exhibit moderate enrichment for pathways including chemokine and cytokine receptors and immune response (**Fig. S6**). All differentially expressed genes and analyses can be queried and downloaded from the open access interactive web resource http://litchi.labtelenti.org.

The RNA sequence data also allowed for estimating the diversity of integration sites during the various time points and upon reactivation. Out of these, we identified 15886 integration events with reads at a junction between host and HIV transcripts. Importantly, the data reflects host-virus splicing events that, while globally identifying the general location of an integration event, do not allow precise mapping. From this we identified 12868 integration events in the host genome across conditions; ranging from 3718 at the earliest time point (*i.e*. 14 days before W0) to 2216 at the latest time point (TCR 24h); 92.5% represented unique integration sites. Overall, these findings indicate minimal clonal bias in the model.

In summary, the stability of the cellular transcriptome profile between weeks 4 and 10 is consistent with maintenance of a resting state of primary CD4+ T cells and limited contribution from viral infection. TCR stimulation creates a cellular environment highly supportive of HIV expression.

### Reactivation with pharmacological and immunological agents

**Figure 5:**
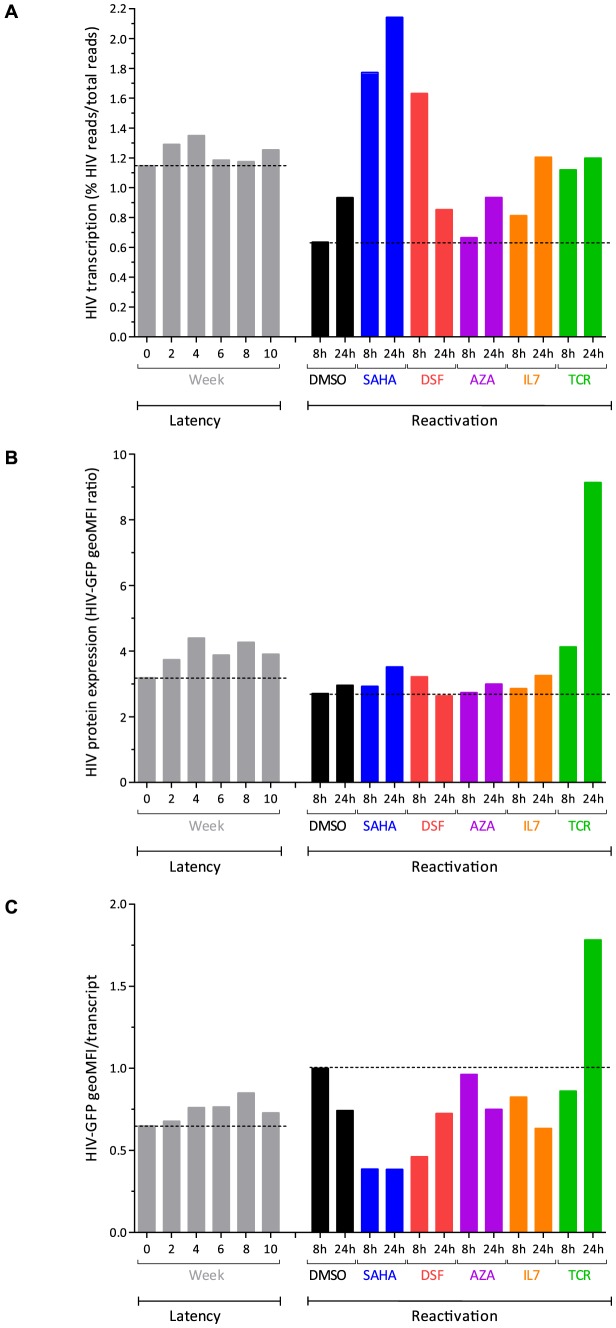
Response to latency reactivating agents. Panel A. Viral transcription (proportion of HIV transcripts to total cellular transcripts). Panel B. Viral expression (GFP) profiles after reactivation with the various compounds. Panel C. HIV GFP protein expression corrected by the proportion of HIV transcript reads in the cell, normalized by DMSO 8 h. The reference lines represent the W0 of latency, and DMSO 8 hour values.

After ten weeks of co-culture on H80, cells were collected and incubated in the presence of various pharmacological (vorinostat [SAHA], disulfiram [DSF], 5′-azacytidine [AZA]), or immunological (interleukin 7 [IL-7]) stimuli, as well as TCR stimulation. We collected RNA for sequencing and cells for FACS analysis at 8 and 24 hours after treatment start. The removal of cells from the feeder cell layer induced a 2-fold reduction of the proportion of HIV to all cellular transcripts (from 1.26% to 0.64% at 8 hours), possibly due to loss of a trophic environment provided by H80 cells (**Fig. 5A**). In contrast, exposure to SAHA resulted in an increase in the proportion of viral transcripts to 1.77% at 8 hours and 2.14% at 24 hours, three times that of DMSO control (**Fig. 5A**). The effect of TCR stimulation on the proportion of HIV transcripts was modest, but cell activation was accompanied by blast morphology and a 2-fold increase in total cellular RNA at 24 hours. Disulfiram had a transient effect, with an increase in the proportion of HIV transcripts to 1.63% at 8 hours. IL-7 had a slower effect with 1.21% at 24 hours. AZA had no detectable impact on viral transcription. The use of the various agents did not result in profound changes in viral vector genome coverage or splicing. However, the use of SAHA was associated with a relative increase in the proportion of unspliced transcripts (**Fig. S3**).

Viral transcription induced by activating agents such as SAHA and disulfiram was not proportionally reflected at the protein level as expression of the viral-encoded GFP remained low (**Fig. 5B**). As previously reported, AZA [24]-[26] alone, or IL-7 [27] had no discernible or minimal effects on viral protein expression. This observation was in contrast to the strong increase in GFP expression induced by TCR stimulation. The divergent response was maximal when estimating GFP translation per transcript (**Fig. 5C**). Ratios were also assessed by considering the production of GFP relative to the amount of single-spliced HIV forms, which have the capability of being translated into GFP. The general profile of transcription, translation and ratios was not modified in this analysis.

**Figure 6:**
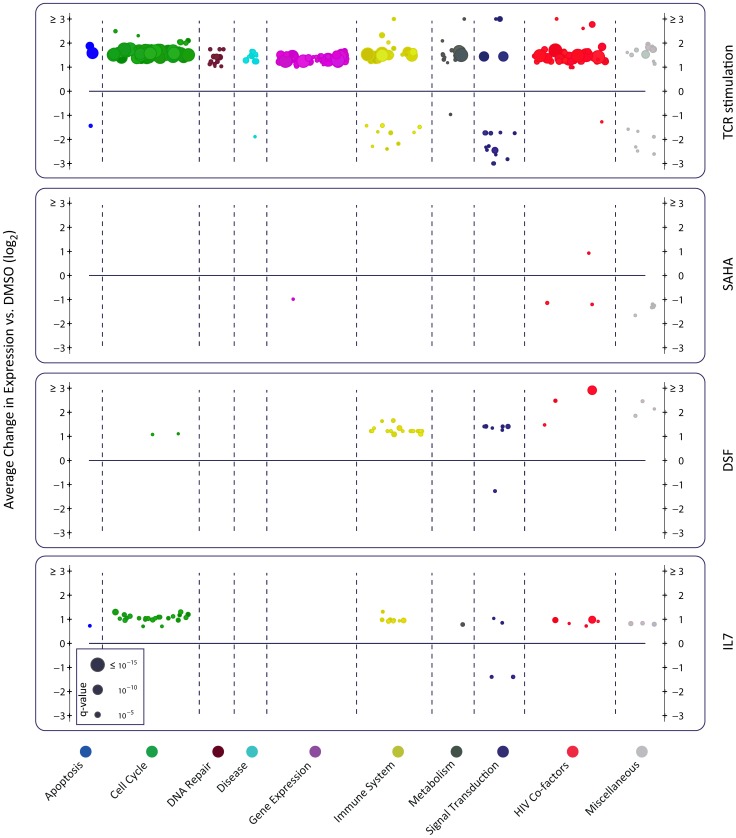
Reactivating agents fail to provide cellular environment necessary for completion of HIV life cycle. Each panel summarizes over-represented pathways among the differentially expressed genes induced by the indicated treatment. Organized under ten major categories, each individual circle represents one enriched pathway in Reactome (see methods). The size is proportional to the adjusted p-value (q-value), and the y-axis corresponds to the average effect of the differentially expressed genes within the reported pathway. Only TCR stimulation using anti-CD3/CD28 and IL-2 leads to upregulation of numerous cellular pathways, as well as upregulation of reported HIV cofactors and HIV related pathways (shown in red). AZA is not shown as no enrichment was observed.

To understand the differential effect of reactivating agents on viral transcription, we compared the transcriptional signatures induced by these agents (**Fig. 6**, and http://litchi.labtelenti.org). TCR stimulation showed a very strong effect involving 3664 upregulated and 3220 downregulated genes, with a strong enrichment signal for upregulation of the cellular machinery (signal transduction, generic gene expression, protein synthesis, and metabolism), and upregulation of genes previously proposed to play a role in HIV biology [28]. In contrast, the various other agents exerted limited influence on the host transcriptome (**Fig. S7**). SAHA induced upregulation of 730 genes and downregulation of 559 genes. Genes involved in immune response and T-cell activation processes were preferentially downregulated by SAHA. Disulfiram showed weak effects on cellular gene expression, with only 132 upregulated genes enriched for HIV cofactors, innate immunity, and apoptosis, and 57 downregulated genes enriched in defense response genes. Very little transcriptomic effects were observed in response to the other reactivating compounds (**Fig. 6** and http://litchi.labtelenti.org).

To confirm the main findings, we repeated the latency and reactivation experiment in the same and on an independent donor. These analyses also included spike-in internal controls for RNA sequencing and used culture supernatant only from H80 feeder cells (without direct cell-cell contact). The data confirmed the reproducibility of the model, and the inability of SAHA to induce viral protein expression in proportion to the increase in viral transcripts (**Fig. S8**). Taken together, these results suggest that, in this model of latency, agents successfully inducing HIV transcription, such as SAHA and disulfiram, do not lead to proportional effects on HIV translation. These agents may not generate a cellular environment that effectively sustains HIV protein expression. In contrast, cell activation upon TCR stimulation creates a permissive environment by systematic activation of the cellular machineries required for viral replication. These results underscore the need to explore strategies that facilitate successful completion of the viral cycle in combination with agents that act primarily on viral transcription.

## Discussion

A number of agents have been identified as latency reverting drugs. SAHA has been extensively studied and brought forward to clinical trials [14], [28], [29]. However, data from Blazkova *et al*. [18], our data, and recent reports at the time this work was under consideration [30]-[32] indicate that SAHA fails to effectively increase particle production *ex vivo*. We tested the ability of SAHA to increase viral production from latently infected CD4+ T cells from virologically suppressed HIV-infected individuals. While SAHA effectively inhibited histone deacetylation, it failed to stimulate particle production as compared to TCR stimulation. Data from the Siliciano laboratory also indicates that reactivation induced by SAHA is not sufficient to trigger a cytopathic effect and cell death [33]. We explored this paradox by using the primary CD4+ T cell model described by Cloyd [19] and Karn [20].

Use of a primary cell model allowed the detailed analysis of the dynamic process of HIV latency and persistence. We examined cellular transcriptional dynamics over 10 weeks and jointly defined cellular and viral expression patterns using RNA sequencing and FACS analysis. In this cellular model, latency appears to be a stable process. However, viral transcripts are continuously present accompanied by residual expression of viral-encoded GFP. We reviewed printed material and figures from various primary models and observed comparable levels of residual GFP or p24 expression that are generally interpreted as background [10], [19], [20], [34], [35] – indeed, the original paper by Tyagi *et al.* identified up to 28.77% of cells expressing low levels of GFP during latency [20]. Upon TCR stimulation, there is near universal induction of GFP expression, generally validating the model. The use of SAHA and disulfiram successfully increased viral transcription; however the reactivated cells failed to produce proportional amounts of viral protein. Overall, these observations underscore the importance of the cellular environment to allow any effect on viral transcription to translate into an efficient purging strategy [1].

We observed a remarkable stability of the cellular transcriptional profile after 4 weeks of co-culture on H80 feeder cells despite the presence of HIV transcripts in the cells. Few cellular genes were differentially expressed between non-infected and infected cells. These genes may represent biomarkers of HIV latency, or markers of the original subset of cells that were successfully transduced. Moreover, the pool of viral transcripts might persist from the original infection or reflect ongoing transcription. Cell-associated viral RNA can also be detected in latently infected resting memory CD4+ T cells in individuals under effective antiretroviral therapy ([14], [36]-[38], and Hu *et al.*, abstract 405, and Fromentin *et al.*, abstract 412, CROI 2014), and in animal models ([39], [40], and Okoye *et al.*, abstract 136LB, CROI 2014). The short half-life of the viral vector-encoded destabilized GFP [10] suggests that some residual translation takes place. Leakiness of the system could be induced by the trophic environment of the experimental system, including secreted factors from the feeder cells, or by the small concentration of IL-2 used in the model. Some level of residual activation has been observed in other primary cell models [41]. Here, however, we only detected negligible levels of cellular activation as assessed by expression of the activation marker IL2Rα (CD25). To better understand the nature of the persisting viral transcripts, we examined the amount and distribution of HIV reads along the viral vector genome, and the pattern of splicing. The proportion of HIV transcripts remained stable at about 1.26% of all cellular transcripts during 10 weeks, without apparent defects in structure. However, the RNA sequencing approach captures polyadenylated RNA; therefore it would not represent coverage of other species of viral transcripts, including non-polyadenylated paused viral transcripts that could be relevant and contribute to the process of latency or reactivation. The leaky system bears some similarities with that of infection of cells belonging to the monocyte/macrophage lineage, which, as discussed by Van Lint *et al.* [41], may not reach complete silencing but maintain a low level of viral replication. Therefore, the model used here may recapitulate both latency and persistence of viral transcription as already observed *in vivo* [1]. However, the experimental system used here has shortcomings. In our hands, the amount of materials generated is limiting, and the use of a complete viral clone is too toxic, which led us to use an attenuated viral vector that only expresses GFP (from *env* ORF), Rev and Tat. The use of such a truncated HIV vector may affect the level of basal HIV transcription and thus the process of latency [42]. Although we are aware of the possibility that, in other types of vectors, specific transcripts and viral proteins other than GFP could be selectively expressed (*e.g.* Gag) [43], the detailed splice analysis militates against major differences in protein expression across the viral genome.

We tested three pharmacological agents to induce viral transcription. SAHA was an efficient inducer, resulting in over 3-fold increase in the proportion of viral transcripts in the cell, consistent with previous reports [14], [16], [44], [45], reviewed in [5]–[7]. The effect of disulfiram was short-lived, with a 2.5-fold induction of viral transcription at 8 hours; also consistent with previous reports [46]. Despite the observed effects on viral transcription, these compounds contributed only minimally to viral protein expression. AZA did not affect viral transcription or translation, consistent with the mode of action of this drug which needs to be incorporated into DNA to be active and prevent methylation. This finding suggests that the cells are not dividing, which again supports the accuracy of our model. In contrast, in cell lines, which are dividing, a longer exposure allows observation of biological effects [24]. In addition, the cellular transcriptional environment was hardly affected by the various drugs, emphasizing that the cells retain a resting state [18], [46], [47]. Use of IL-7 contributed minimally to enhanced viral transcription or translation, despite exerting more pronounced effects on cellular transcription, consistent with recent reports on the use of this cytokine *in vivo* [48]. In contrast, the effect on the transcriptome and the cellular environment created by TCR stimulation strongly favored translation.

Our data raise the possibility of post-transcriptional block as one of the mechanisms of HIV latency. This is consistent with studies from the Fauci laboratory that indicated that resting CD4+ T cells from aviremic patients did not produce quantifiable cell-free virions despite the presence of cell-associated HIV-1 RNA [18], [49]. Proposed mechanisms underlying post-transcriptional blocks include splicing defects [43], inhibition of nuclear RNA export [50], inhibition of expression of viral proteins in a codon-usage-dependent manner [51], and inhibition of HIV translation by microRNAs [41], [52]. Our current work, and results from Iglesias-Ussel *et al.* [53] suggest that latently infected cells may have multiple biochemical and metabolic blocks that are not completely released by some of the reactivating agents currently being evaluated.

Cell line models and primary CD4+ T cell models have advanced the understanding of basic mechanisms of HIV transcriptional latency and served to screen for latency reversing agents. In particular, cell lines highlighted the role of multiple *cis*- and *trans*-acting factors in the regulation of basal viral transcription [5], [7]. Primary cell models provided a broader view on the complexity of latency and variability in response to reactivating agents [13], [54]. Our work on the dynamics of latency and reactivation using a primary cell model underscores the existence of barriers beyond transcriptional silencing – thus helping explain clinical trials and *ex vivo* data that identify persistent viral transcription but impaired viral protein expression and particle production. Overcoming post-transcriptional blocks may thus require additional interventions.

## Materials and Methods

### Viral production from latently infected cells *ex vivo*

We collected 25 ml of total blood from HIV-infected individuals participating in the Swiss HIV Cohort Study (http://www.shcs.ch) with controlled viremia (**Table S1**). Resting CD4+ T cells were purified by Ficoll gradient separation followed by negative selection and magnetic separation using the human CD4+ T Cell enrichment kit supplemented with anti-HLA-DR, anti-CD25 and anti-CD69 (Stem Cell Technologies). Cells were resuspended in Opti/FCS/IL-2, split in three wells (approximately 1 million/ml/well) containing DMSO, 0.5 µM SAHA, or TCR stimulation as described below. After 1, 2 or 4 days of incubation, cell supernatant was collected and assessed for viral presence in a COBAS AMPLICOR analyzer (Roche), with a limit of detection of 20 unspliced RNA copies/ml.

### Histone immunoblotting

Resting CD4+ T cells from HIV-infected individuals were isolated and treated for 24 h with DMSO, SAHA or TCR stimulation as indicated above. Histone extraction was performed according to Abcam's protocol. Briefly, cells were washed twice with ice-cold PBS, resuspended in Triton Extraction Buffer (PBS–0.5% Triton X100–protease inhibitors–0.02% NaN_3_) at 10^7^ cells/ml and incubated 10′ on ice to allow cell lysis. Cells were centrifuged at 380 g for 10′ at 4°C and washed once with Triton Extraction Buffer. After centrifugation, the pellet was resuspended at 4×10^7^ cells/ml in 0.2N HCl and incubated over night at 4°C to allow for histone extraction. Samples were centrifuged at 380 g for 10′ at 4°C and the histone-containing supernatant was collected and protein concentration was measured using Qubit Protein Assay (Life Technologies) following manufacturer's protocol. Histone extracts (1 µg) were separated on an 8–16% SDS-polyacrylamide gel, transferred to a nitrocellulose membrane and processed for immunoblotting. Briefly the membranes were washed with PBS, blocked for 2 h at RT in PBST-milk (PBS/0.2% Tween-5% non fat dry milk), incubated over night with anti-histone antibodies (1∶1000) at 4°C, washed three times for 10′ in PBST, incubated with swine anti-rabbit-HRP (Dako P-0217, 1∶2000) for 2 h at RT, washed again and revealed using Luminata Crescendo Western HRP substrate (Millipore). Primary antibodies were rabbit polyclonal from Abcam: anti-H3K27Ac (ab4729), anti-H3K9Ac (ab10812), anti-H3 (ab1791).

### Primary cell model

Primary CD4+ T cells were purified by Ficoll gradient separation followed by negative selection and magnetic separation using the human CD4+ T Cell Isolation kit II (Miltenyi Biotec) (**Fig. S1**). Cells were activated using CD3/CD28 co-stimulation in presence of IL-2 (mimicking TCR stimulation) as described previously [10]. Briefly, anti-CD3 antibodies (10 µg) were plated in 1 ml PBS per well of a 6-well plate and incubated for 1–2 h at 37°C. Wells were then washed once with 3 ml of PBS and filled with 10^6^ cells/ml of primary CD4+ T cells supplemented with 1 µg/ml anti-CD28 antibodies in Optimizer CTS T-Cell expansion SFM culture medium containing 5% heat-inactivated fetal calf serum (FCS) and 100 U/ml human recombinant IL-2 (Opti/FCS/IL-2 culture medium). Three days post-stimulation, cells were collected, washed and resuspended at 10^6^ cells/ml in Opti/FCS/IL-2 for infection with an HIV-based vector (NL4-3-Δ6-drEGFP/CXCR4; a kind gift from R.F. Siliciano). This HIV vector uses a CXCR4 tropic HIV envelope for entry, contains functional *tat* and *rev* genes, as well as a *gfp* reporter gene containing a PEST sequence (yielding a protein half time of ∼6 hours); this vector contains mutations (stop codons, frameshift or deletion) in *gag*, *vif*, *vpr*, *vpu*, *env* and *nef* and is thus less cytotoxic for the infected cell, thereby promoting latency in a higher proportion of infected cells [10]. HIV particles were produced by transfection of HEK293T cells as previously described [22]. Infection was carried out by spinoculation for 3 hours at 25°C and at 1500 g using 50 µg p24 equivalent HIV vector/10^6^ cells in presence of 5 µg/ml polybrene. After spinoculation, cells were washed and resuspended at 10^6^ cells/ml in Opti/FCS/IL-2. Forty-eight hours post-infection, cells were resuspended in PBS/FCS (to eliminate the interference of phenol red) and sorted by FACS according to GFP expression, yielding a typical purity of >98%. FACS sorting was performed with a Mo-Flo Astrios instrument. Upon sorting, successfully infected GFP+ cells were further processed to investigate HIV latency.

Establishment and maintenance of HIV latency was mostly carried out as previously described [19], [20] (**Fig. S1**). Infected GFP+ cells stimulated using anti-CD3 and anti-CD8 antibodies in presence of IL-2 as described above were expanded in Opti/FCS/IL-2 for ∼3 weeks, typically allowing ∼2 log of cell multiplication. At this time (week 0), cells were washed, resuspended in R-10/IL-2 (RPMI-1640 culture medium supplemented with 10% heat-inactivated FCS and 40 U/ml IL-2) and added to an adherent H80 feeder cell layer. The H80 human brain tumor cell line, known to promote *ex vivo* survival of primary CD4+ T cells, were prepared by plating 10^6^ cells/25 cm2 flask (T25) or 3×10^6^ cells/75 cm2 flask (T75) the day before starting co-culture with primary CD4+ T cells. CD4+ T cells were resuspended in R-10/IL-2 and added on top of the feeder cell layer, in 16 ml R-10/IL-2 in T75 if 30-100×10^6^ primary CD4+ T cells or in 5 ml R-10/IL-2 if >30×10^6^ cells. Culture medium was changed three times a week by renewing half of it. Every two weeks, CD4+ T cells were collected and transferred onto a new feeder cell layer flask. During this transfer, cell samples were collected for analyses (see below). The co-culture of primary CD4+ T cells with H80 feeder cells was carried over a period of 10 weeks to allow CD4+ T cells to revert to a resting phenotype and to ensure entry into and maintenance of latency. Collected samples were used for: (i) assessing the expression of virally-encoded GFP and of the marker of activation IL2Rα (CD25) by FACS analysis, and (ii) assessing dynamic changes of the cellular and viral transcriptomes using mRNA-Seq. This analysis allowed for (a) following the cellular dynamic process from activated to resting state at the transcriptional level in non-infected as well as infected cells, (b) highlighting genes differentially expressed in HIV-infected cells as compared to mock cells, and (c) measuring concomitant viral transcriptional activity in resting CD4+ T cells. The latency process was also repeated using H80 supernatant as alternative to cell-to-cell contact conditions.

### Reactivation

We included pharmacological and immunological agents that affect HIV transcriptional activity by different mechanisms of action and that are currently under clinical consideration. Specifically, we used a histone deacetylase inhibitor (vorinostat or SAHA, 0.5 µM), a DNA methylation inhibitor (5′-azacytidine, 1 µM), an alcohol dehydrogenase inhibitor (disulfiram, 0.5 µM). All drugs were suspended in dimethyl sulfoxyde (DMSO), thus DMSO was used as a negative control (of note, final DMSO concentration was 0.0033% corresponding to 1∶30,000 dilution). We also used immunological stimulation by interleukin-7 cytokine (100 ng/ml), and a full TCR stimulation using anti-CD3/anti-CD28 antibodies in presence of interleukin-2. At 8 h and 24 h after treatment, cells were analyzed for GFP expression by FACS and for transcriptome composition as described below.

### RNA sequencing

Cell samples were stored in RNALater at the time of collection. Once all samples, from week 0 to week 10, were collected, total RNA extraction was performed using Illustra RNAspin mini isolation kit (GE Healthcare) and further processed for mRNA-Seq library preparation (TruSeq RNA sample prep kit, Illumina – that starts with capture of polyA-containing transcripts), followed by cluster generation (TruSeq single-end cluster generation kit, Illumina) and high-throughput sequencing on Illumina HiSeq2000 at the Genomics Technology Facility, University of Lausanne. Addition of RNA spike-ins were also added according to manufacturer's instructions (ERCC Exfold RNA Spike-in Mixes, Life Technologies and Loven *et al.* [55]). We obtained about 100 mio single end reads of 100 nucleotides for each sample. The sequencing reads were cleaned before alignment in order to improve the accuracy of downstream analyses. The cleaning steps included the removal of (i) Illumina's adapter (if present at the 3' end of the read) with cutadapt v0.9.5 [56], (ii) low quality (PHRED score<6) nucleotides at the 3′ or 5′ end of the reads, (iii) reads with mean PHRED score lower than 20 and (iv) polyA tails with prinseq v0.17 [57]. Only reads of 30 nucleotides or longer after trimming were kept for further analyses. The cleaned reads were aligned to the reference genome with TopHat v2.0.6 [58] using the ensembl gene GRCh37 release 68 annotation file. The reference genome was built by concatenating the human genome (hg19) and the HIV vector sequence. The number of reads per gene was extracted with a modified version of HTSeq-count v0.5.3 (http://www-huber.embl.de/users/anders/HTSeq/doc/count.html) using the same annotation file and considering the whole HIV vector as a single gene. The modifications allowed multiply aligned reads to be weighted accordingly. Ambiguous reads were randomly attributed to one of the genomic regions they aligned to. To identify integration sites we used Tophat fusion with bowtie1 and default parameters.

### Variance stabilization

Two-way analysis of variance and principle component analysis (PCA) were used for quantitative assessment of the transcriptome data presented in **Fig. 4**. In order to fulfill the underlying assumptions of these models, we conservatively discarded lowly expressed genes to minimize heteroscedasticity due to shot noise. Retaining genes expressing at least 100 reads in more than half of the samples yielded 14,513 genes. The resulting dataset was smoothed by adding pseudocounts (10 extra reads per gene), and log-transformed for variance stabilization.

### Differential expression tests

Differential expression tests were performed assuming negative-binomially distributed read counts using the Bioconductor package DESeq [29]. Paired-sample differential expression tests were performed using generalized linear models and dispersions were estimated using the Cox-Reid-adjusted maximum likelihood estimator. Lowly expressed genes were discarded after estimating dispersion. All p-values were corrected using a false discovery rate of 5% (Benjamini-Hochberg procedure [59]).

### Pathway enrichment analysis and visualization

Enrichment analyses were performed using Fisher's exact test to detect overrepresentations of functional classes, regulatory motifs (among miRNA and transcription factor targets), physical locations on chromosomes, and HIV-related pathways, proteins and co-factors as described previously [17]. HIV co-factors include genes in HIV related pathways from the Reactome pathways database Ver.40, genes identified in previous siRNA studies [60]–[63], and genes encoding protein interaction partners for each viral protein ([28] and http://mint.bio.uniroma2.it/virusmint). All p-values were corrected using a false discovery rate of 5%.

For visualization in **Fig. 6**, we only considered enrichment hits from Reactome pathways and HIV co-factors databases. The major pathway categories were chosen from maximal pathways, (*P* ***_i_***), among all the reported unique enrichment hits pooled over all reactivating agents. In order to choose the most representative pathway categories, we defined the asymmetric coverage score of the *i* ^th^ pathway, *P_i_,* over the *j* ^th^ pathway, *P_j_,* as *m_i_*_,*j*_ = |*P_i_* ∩ *P_j_*|/|*P_j_*| and the total coverage of the *i* ^th^ pathway as *S_i_* = ∑*_j_ m*_i,j_. The set of representative categories was built by those with the highest total coverage score *S_i_*. We used the nine top categories plus a pseudo-pathway called “miscellaneous” for the visualization. Each pathway was assigned to the major category that holds the highest coverage score over it. Subsequently, the pathways that are not covered more than 95% in any of the chosen major categories are assigned to the miscellaneous category.

### Data and materials availability

All data, including differentially expressed genes and enrichment analyses, are freely available in interactive mode or for downloading at http://litchi.labtelenti.org.

### Bioinformatics of viral transcriptome

Two splice junction aligners were used to identify HIV splicing events: Tophat v2.0.6 and STAR v2.3.0 [64]. All acceptor-donor pairs were retrieved and their proportion calculated. Both splice junction aligners gave similar and consistent results. The HIV transcriptome profile was assessed using the Python package HTSeq (http://www-huber.embl.de/users/anders/HTSeq/doc/overview.html). Coverage was normalized by the sample size factor estimated using DESeq. To calculate the percentage of HIV transcripts among all transcripts, we compared the gene length normalized counts using the length of the longest annotated transcript (isoforms with intron retention were not considered).

The main viral RNA forms were estimated as follows. The number of reads overlapping the D1 junction corresponds to unspliced reads (US, non-spliced in D1). The number of reads aligning to the left of D1 and broken at D1 (up to a different HIV position) corresponds to reads spliced in D1 and thus belongs to singly spliced or multiply spliced HIV RNA (MS+SS). The number of reads overlapping the D4 junction corresponds to singly spliced or unspliced HIV transcripts (US+SS). The number of reads aligning to the left of D4 but broken at D4 (up to different HIV position) corresponds to reads spliced in D4 and thus belong to multiply spliced transcripts (MS). Finally, the total proportion of reads (100%) is US+MS+SS, and SS can be obtained as SS = 100%–US–MS.

## Supporting Information

Figure S1 **Experimental design.** Cellular model.
(PDF)

Figure S2 **FACS analysis of HIV-encoded GFP expression of representative cell samples.** Panel A. CD4+ T cells were analyzed by FACS to assess the geometric mean fluorescence intensity of GFP expression (FL1-H channel). The histogram plot shows uniform populations (single peaks) for uninfected controls (W10+24 h DMSO, dotted grey line), latently infected cells (W10, red solid line), latently infected cells upon 24h reactivation with DMSO (black solid line), SAHA (blue solid line) or TCR stimulation (green solid line). Data revealed a shift in intensity of TCR-stimulated cells but not SAHA-treated cells as compared to the corresponding DMSO control. Panel B. Dot plots of FSC/SSC or FL1/FL2 of CD4+ T cells, either mock or HIV-infected, at week 10 post co-culture on H80 (W10) and 24 h post TCR stimulation (TCR). FSC/SSC dot plots shows differences in cell size and complexity between W10 and TCR, likely representative of resting CD4+ T cells and activated CD4+ T cells respectively. FL1/FL2 dot plots shows highly expressing GFP cells in R7 region upon TCR stimulation.
(PDF)

Figure S3 **Features of HIV transcription under several reactivation agents.** Panel A. Distribution of HIV reads along the vector genome; each panel compares one agent against DMSO as control. On the top is depicted the viral vector genome used. TSS: transcription start site; D: splice donor; A: splice acceptor. Reads mapping to the LTR are equally assigned to 5′ and 3′ ends. Panel B. Pattern of splicing for the main viral RNA forms: genomic unspliced full-length viral RNA (US, blue), singly spliced RNAs without the Gag-Pol major intron (SS, green; spliced in D1 but not in D4), and multiply spliced subgenomic mRNAs (MS, red; spliced in D1 and in D4).
(PDF)

Figure S4 **Features of HIV transcription under several reactivation agents.** Detailed assessment of donor-acceptor splice junction usage and graphical representation. D: splice donor; A: splice acceptor.
(PDF)

Figure S5 **Principal component analysis of latency and TCR stimulation compared to H80 feeder cells.** The transcriptome of H80 feeder cells (two replicates) is distinct. There is no evidence for contamination of primary CD4+ T cells during the process of latency. Upon TCR stimulation, the CD4+ T cells are removed from the H80 feeder cells.
(PDF)

Figure S6 **Pathway enrichments for the differentially expressed genes during HIV latency.** Each panel summarizes over-represented pathways among the differentially expressed genes induced by viral presence. Organized under nine major categories, each individual circle represents one enriched pathway in Reactome (see methods). The size is proportional to the adjusted p-value (q-value), and the y-axis corresponds to the average effect of the differentially expressed genes within the reported pathway.
(PDF)

Figure S7 **Principal component analysis of modifications in the transcriptome upon exposure to the various reactivating agents.** The transcriptome of CD4+ T cells exposed to the various reactivating agents cluster with that of mock and of latently infected cells (W4 to W10), suggesting a minimal impact of those compounds on the cell. Panel A shows the transcriptome data in the context of latency phase and full cell activation by TCR stimulation. Panel B shows a PCA analysis on the large cluster of cells exposed to reactivating agents. The PCA of the cluster reveals small compound- and HIV-specific transcriptome differences compared to W10 infected and uninfected CD4+ T cells.
(PDF)

Figure S8 **Validation and reproducibility of the model.** Panel A. Viral transcription (viral-encoded GFP transcripts normalized by internal control and by baseline DMSO values) upon SAHA or TCR stimulation on two donors. Panel B. Viral expression (GFP MFI) profile after reactivation with SAHA or TCR on two donors. Panel C. Principal component analysis of modifications in the transcriptome upon exposure to the various reactivating agents. An additional experiment was performed to include cellular samples prior co-culture with H80 cell supernatant (mD14, mD12 and mD9 corresponding to cells collected 14, 12 and 9 days before W0 respectively). This additional set of CD4+ T cell transcriptomes recapitulates the entry, latency and TCR reactivation that were already observed. Exposure to H80 did not require cell-to-cell contact as the experiment used only filtered H80 cell culture supernatant. These additional cellular samples also included RNA spike-in controls (spike) to control for RNA content differences; normalization data using library size or RNA spike-in were similar.
(PDF)

Table S1 **Characteristics of HIV-infected individuals included in the** ***ex vivo*** **activation study.**
(PDF)
