# Supplementary figures and images for: Dynamics of HIV Latency and Reactivation in a Primary CD4+ T Cell Model
Source: PLoS Pathog. 2014 May 29;10(5):e1004156. doi: 10.1371/journal.ppat.1004156 (PMC4038609; doi:10.1371/journal.ppat.1004156)

## Infection process:

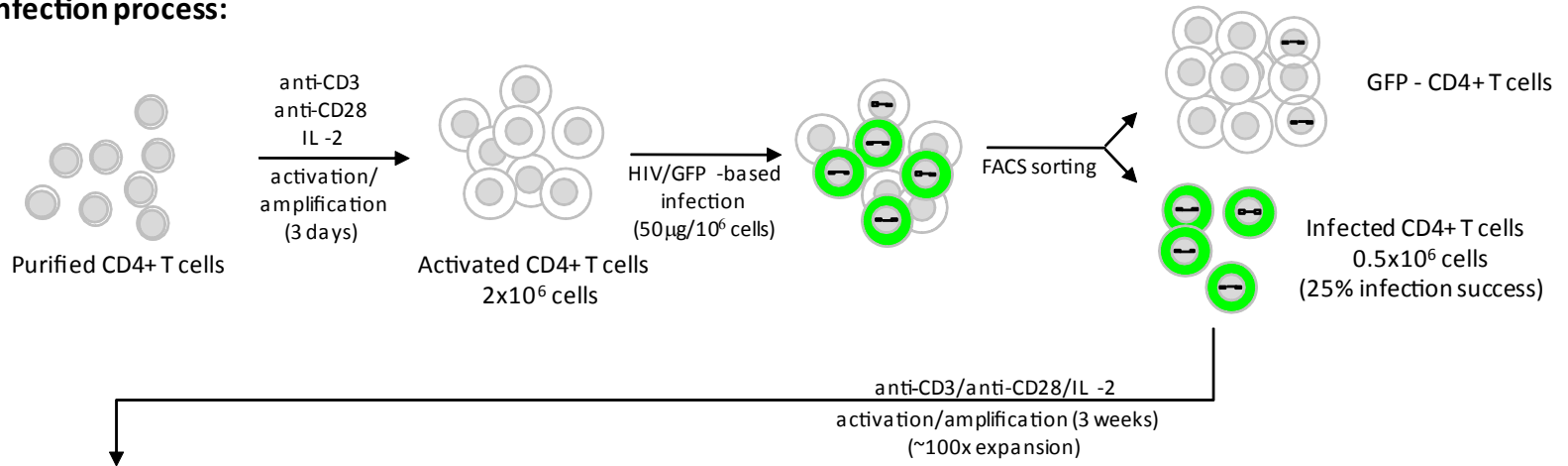

## Latency process:

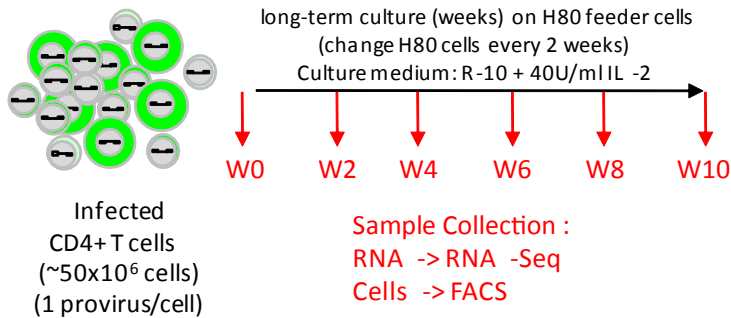

## Reactivation process:

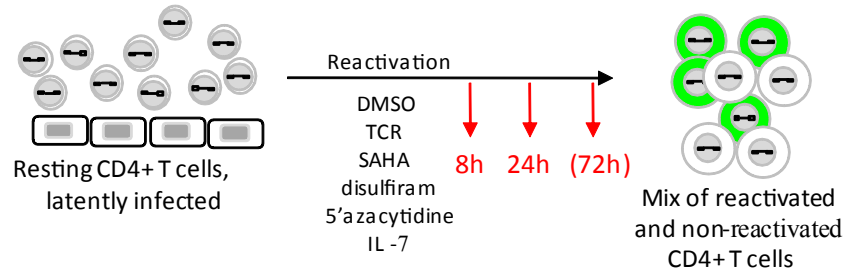

Supplement: Figure S1 — Experimental design. Cellular model. (PDF) [file ppat.1004156.s001.pdf]

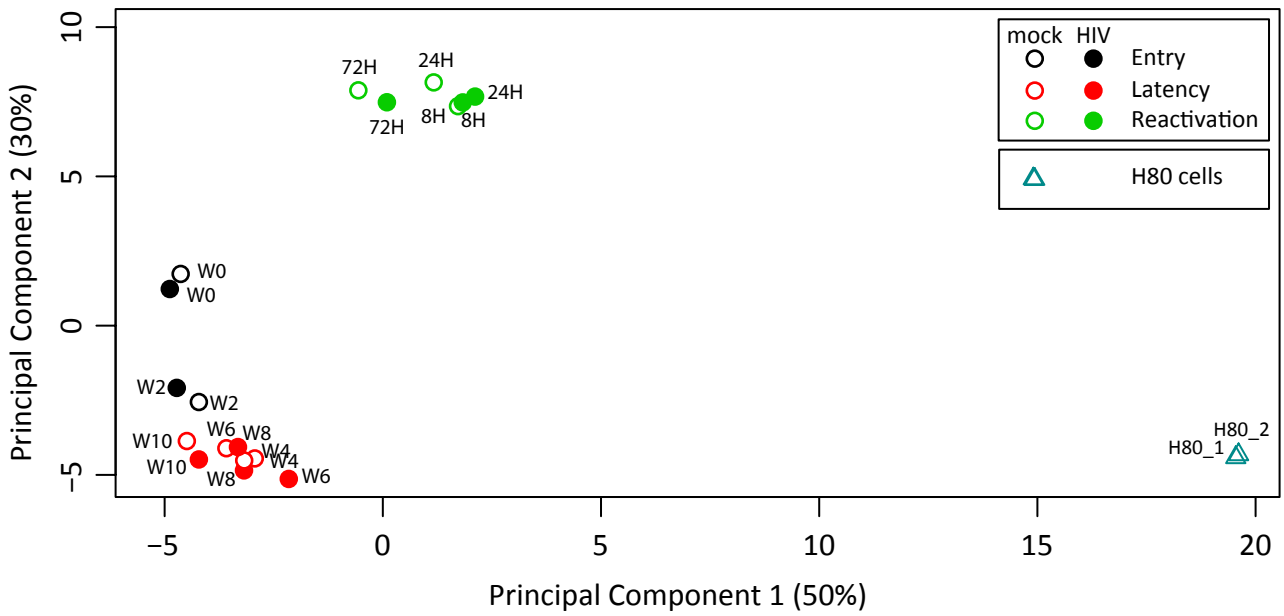

Supplement: Figure S5 — Principal component analysis of latency and TCR stimulation compared to H80 feeder cells. The transcriptome of H80 feeder cells (two replicates) is distinct. There is no evidence for contamination of primary CD4+ T cells during the process of latency. Upon TCR stimulation, the CD4+ T cells are removed from the H80 feeder cells. (PDF) [file ppat.1004156.s005.pdf]

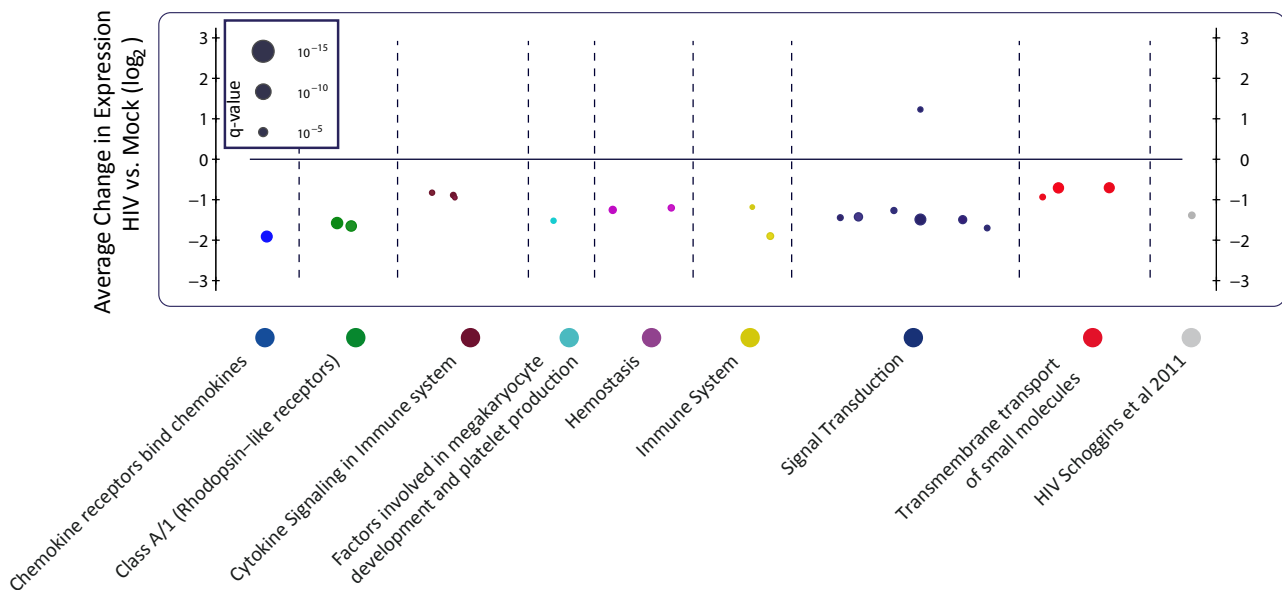

Supplement: Figure S6 — Pathway enrichments for the differentially expressed genes during HIV latency. Each panel summarizes over-represented pathways among the differentially expressed genes induced by viral presence. Organized under nine major categories, each individual circle represents one enriched pathway in Reactome (see methods). The size is proportional to the adjusted p-value (q-value), and the y-axis corresponds to the average effect of the differentially expressed genes within the reported pathway. (PDF) [file ppat.1004156.s006.pdf]

**A**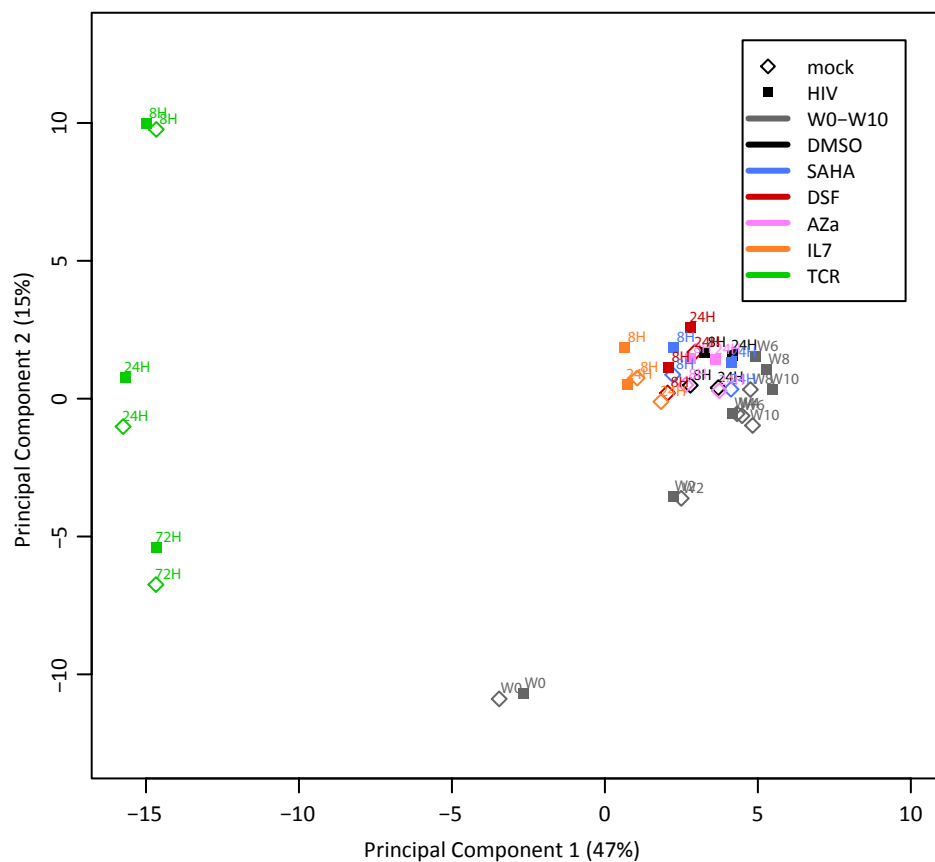**B**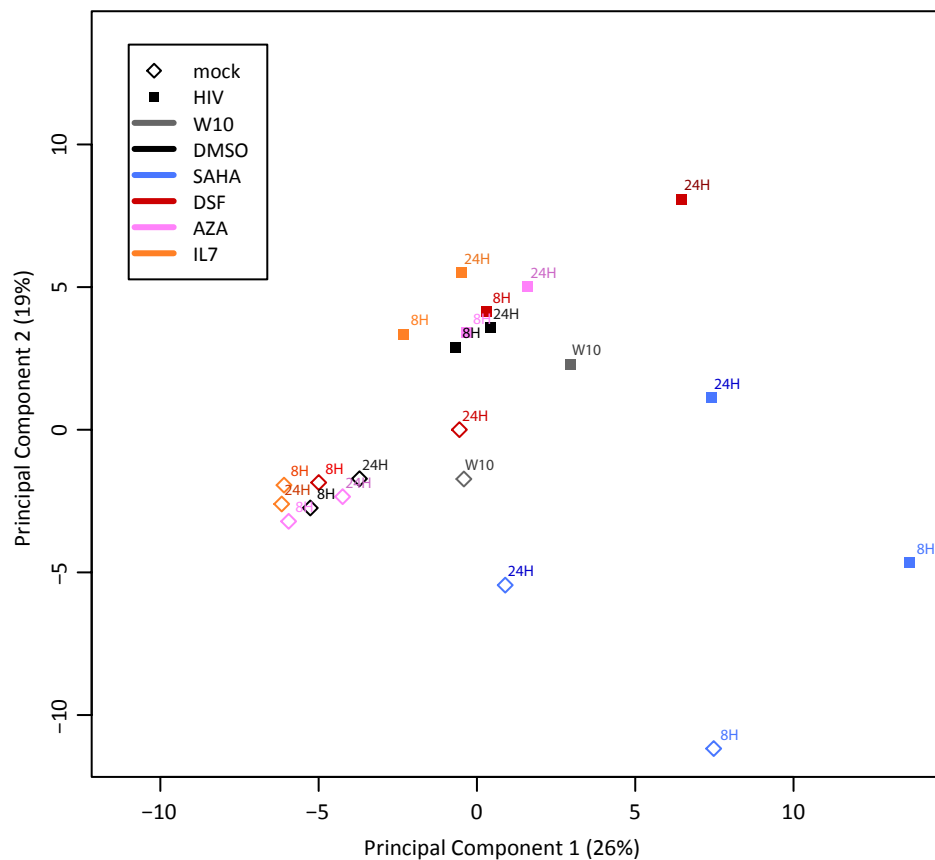

Supplement: Figure S7 — Principal component analysis of modifications in the transcriptome upon exposure to the various reactivating agents. The transcriptome of CD4+ T cells exposed to the various reactivating agents cluster with that of mock and of latently infected cells (W4 to W10), suggesting a minimal impact of those compounds on the cell. Panel A shows the transcriptome data in the context of latency phase and full cell activation by TCR stimulation. Panel B shows a PCA analysis on the large cluster of cells exposed to reactivating agents. The PCA of the cluster reveals small compound- and HIV-specific transcriptome differences compared to W10 infected and uninfected CD4+ T cells. (PDF) [file ppat.1004156.s007.pdf]

**A**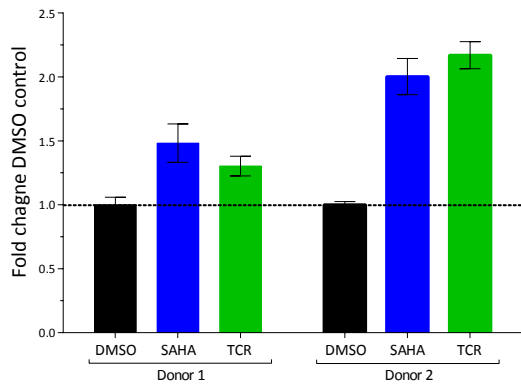**B**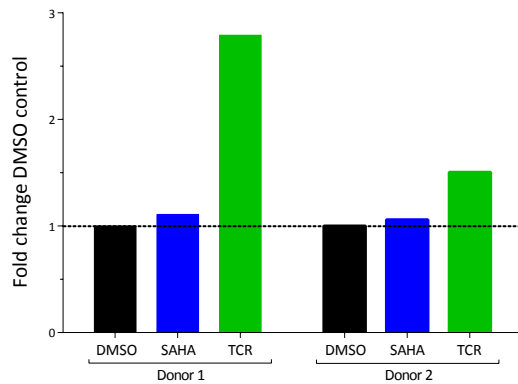**C**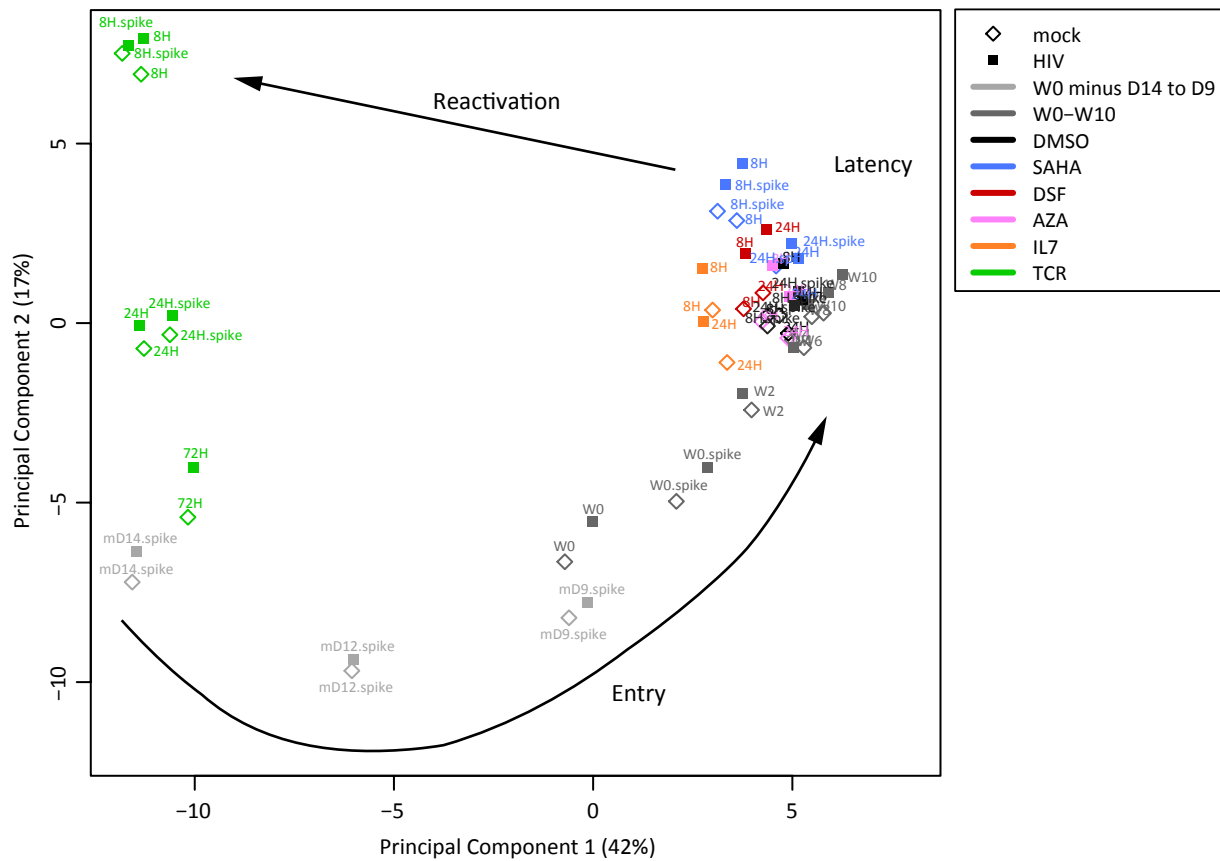

Supplement: Figure S8 — Validation and reproducibility of the model. Panel A. Viral transcription (viral-encoded GFP transcripts normalized by internal control and by baseline DMSO values) upon SAHA or TCR stimulation on two donors. Panel B. Viral expression (GFP MFI) profile after reactivation with SAHA or TCR on two donors. Panel C. Principal component analysis of modifications in the transcriptome upon exposure to the various reactivating agents. An additional experiment was performed to include cellular samples prior co-culture with H80 cell supernatant (mD14, mD12 and mD9 corresponding to cells collected 14, 12 and 9 days before W0 respectively). This additional set of CD4+ T cell transcriptomes recapitulates the entry, latency and TCR reactivation that were already observed. Exposure to H80 did not require cell-to-cell contact as the experiment used only filtered H80 cell culture supernatant. These additional cellular samples also included RNA spike-in controls (spike) to control for RNA content differences; normalization data using library size or RNA spike-in were similar. (PDF) [file ppat.1004156.s008.pdf]
