# Supplementary material for: Dynamics of HIV Latency and Reactivation in a Primary CD4+ T Cell Model
Source: PLoS Pathog. 2014 May 29;10(5):e1004156. doi: 10.1371/journal.ppat.1004156 (PMC4038609; doi:10.1371/journal.ppat.1004156)
Supplement: Figure S2 — FACS analysis of HIV-encoded GFP expression of representative cell samples. Panel A. CD4+ T cells were analyzed by FACS to assess the geometric mean fluorescence intensity of GFP expression (FL1-H channel). The histogram plot shows uniform populations (single peaks) for uninfected controls (W10+24 h DMSO, dotted grey line), latently infected cells (W10, red solid line), latently infected cells upon 24h reactivation with DMSO (black solid line), SAHA (blue solid line) or TCR stimulation (green solid line). Data revealed a shift in intensity of TCR-stimulated cells but not SAHA-treated cells as compared to the corresponding DMSO control. Panel B. Dot plots of FSC/SSC or FL1/FL2 of CD4+ T cells, either mock or HIV-infected, at week 10 post co-culture on H80 (W10) and 24 h post TCR stimulation (TCR). FSC/SSC dot plots shows differences in cell size and complexity between W10 and TCR, likely representative of resting CD4+ T cells and activated CD4+ T cells respectively. FL1/FL2 dot plots shows highly expressing GFP cells in R7 region upon TCR stimulation. (PDF) [file ppat.1004156.s002.pdf]

A

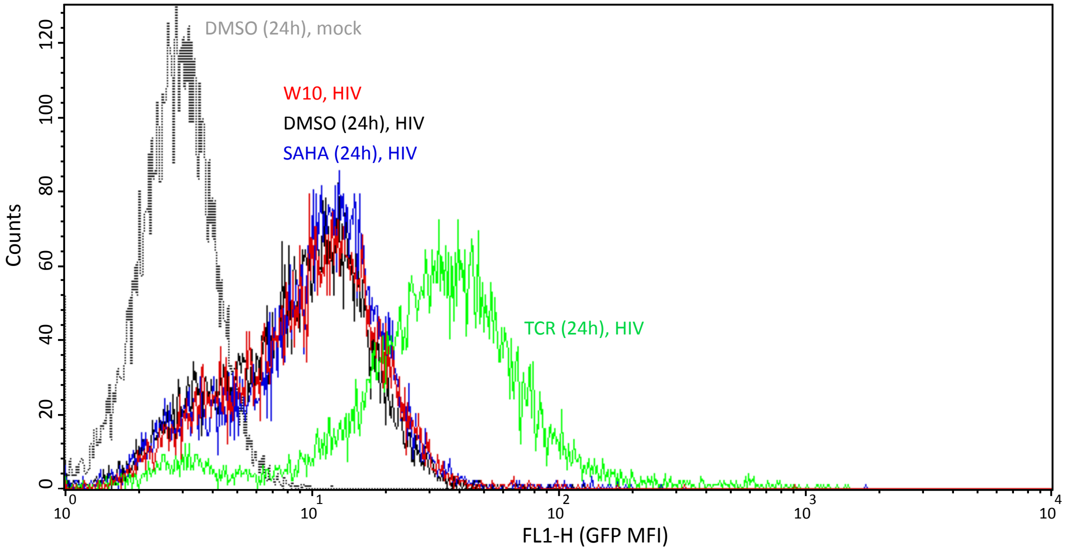

B

mock, W10

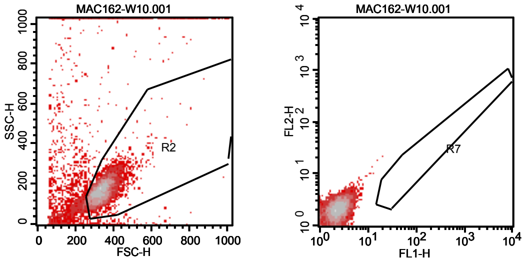

Region	Events	% Gated	% Total	X Geo Mean
R2	10281	100.00	77.06	2.35
R7	0	0.00	0.00	***

mock, TCR 24h

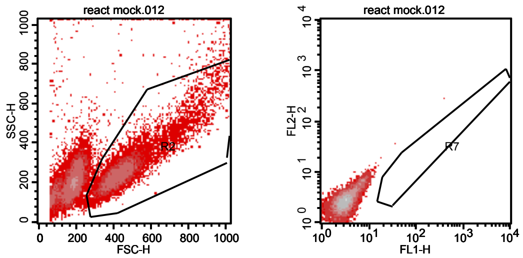

Region	Events	% Gated	% Total	X Geo Mean
R2	10140	100.00	42.15	3.36
R7	0	0.00	0.00	***

HIV, W10

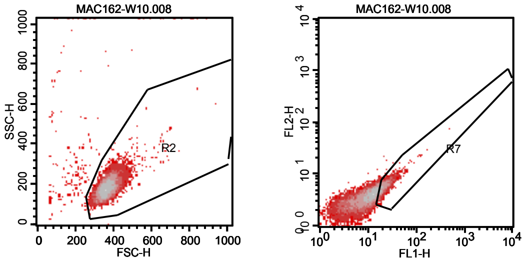

Region	Events	% Gated	% Total	X Geo Mean
R2	10011	100.00	98.27	9.15
R7	1581	15.79	15.52	23.00

HIV, TCR 24h

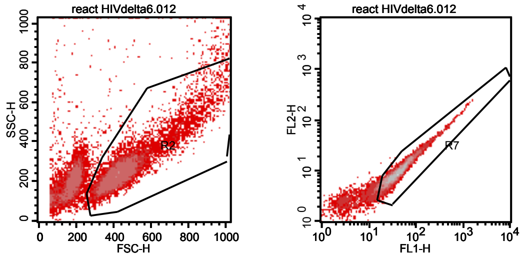

Region	Events	% Gated	% Total	X Geo Mean
R2	10148	100.00	62.49	30.78
R7	8283	81.62	51.00	41.94
