# Supplementary material for: Dynamics of HIV Latency and Reactivation in a Primary CD4+ T Cell Model
Source: PLoS Pathog. 2014 May 29;10(5):e1004156. doi: 10.1371/journal.ppat.1004156 (PMC4038609; doi:10.1371/journal.ppat.1004156)

Splice Junction	Position	% of spliced HIV transcript																	
		Week 0	Week 2	Week 4	Week 6	Week 8	Week 10	DMSO 8H	DMSO 24H	SAHA 8H	SAHA 24H	DISU 8H	DISU 24H	AZA 8H	AZA 24H	IL7 8H	IL7 24H	CD3/CD28 8H	CD3/CD28 24H
D4_A7	6044-8278	23.46	26.76	26.3	27.11	27.31	29.57	27.31	26.96	23.01	25.45	26.26	30.24	24.59	25.41	25.44	25.39	28.45	26.73
D1_A1	743-4913	13.94	14.19	14.47	14.72	15.2	13.61	14.69	13.72	17.11	16.71	14.44	16.24	15.18	14.38	15.42	14.28	14.07	13.02
D1_A2	743-5390	12.54	12.05	10.95	11.25	10.72	11.35	10.75	10.29	12.6	10.38	12.37	8.67	11.68	10.98	12.42	11.81	13	13.95
D1_A5	743-5976	13.06	16.15	16.46	16.73	17.28	14.26	14.41	14.79	14.83	16.37	14.4	15.43	15.76	16.27	14.76	15.03	9.45	9.99
D3_A5	5463-5976	6.95	8.12	8.4	7.98	7.56	8.84	9.1	9.25	8.07	7.98	9.42	8.09	8.81	8.63	8.98	8.85	8.98	8.95
D2_A5	4962-5976	3.34	3.9	4.72	4.25	4.42	4.39	4.63	4.62	4.32	4.34	4.54	4.7	4.46	4.09	4.31	3.98	3.95	3.27
D2_A2	4962-5390	2.68	2.94	3.09	3.15	2.9	3.87	4.1	4.04	3.47	3.47	4.42	3.89	3.63	3.55	3.41	3.53	3.87	3.82
D4_A6new1	6044-6306	3.25	1.83	1.3	1.11	1.25	1.23	1.46	1.83	1.26	1.33	1.62	0.99	1.26	1.36	1.01	1.32	2.5	4.1
D2_A3	4962-5777	3.32	2.36	2.69	2.53	2.59	2.81	2.5	2.82	2.83	2.65	2.02	2.89	2.6	2.91	2.22	2.63	2.4	1.93
D1_A3	743-5777	5.19	1.9	1.96	1.98	1.92	1.63	1.74	1.94	2.45	1.94	1.33	1.7	2.05	2.27	1.86	2.25	2.36	2.61
other		12.04	9.51	9.36	8.93	8.63	8.23	9.05	9.49	9.74	9.02	9.01	6.94	9.77	9.95	9.85	10.77	10.75	11.35

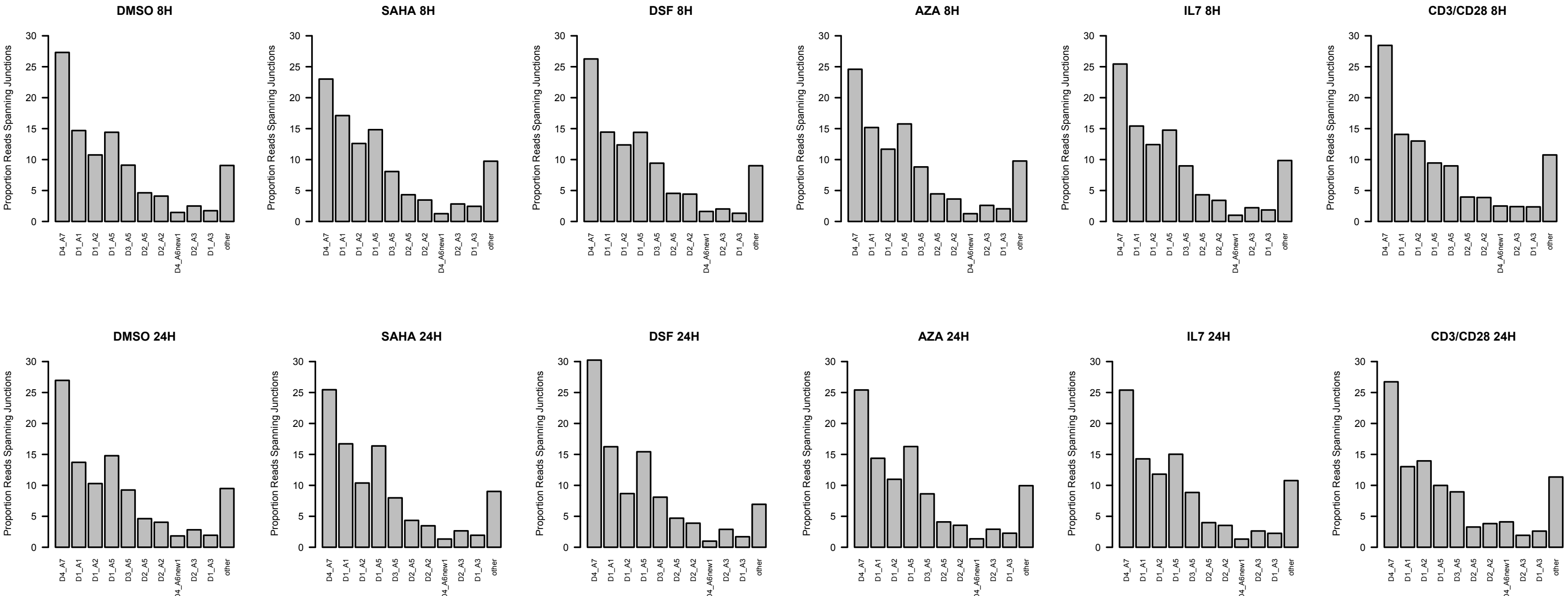

Supplement: Figure S4 — Features of HIV transcription under several reactivation agents. Detailed assessment of donor-acceptor splice junction usage and graphical representation. D: splice donor; A: splice acceptor. (PDF) [file ppat.1004156.s004.pdf]
