# Supplementary material for: Dynamics of HIV Latency and Reactivation in a Primary CD4+ T Cell Model
Source: PLoS Pathog. 2014 May 29;10(5):e1004156. doi: 10.1371/journal.ppat.1004156 (PMC4038609; doi:10.1371/journal.ppat.1004156)
Supplement: Table S1 — Characteristics of HIV-infected individuals included in the ex vivo activation study. (PDF) [file ppat.1004156.s009.pdf]

SHCS ID	Current			History				Date of HIV diagnosis	Gender	Age	Sample ID (Fig.S8)
	Viremia	CD4	Treatment	MONTHS <50 copies	Nb of blips 20-49	Nb of Blips(50-400)	Baseline viremia before treatment				
52193	<20	591	TDF,FTC,ATV,RTV	75	4	1	5590	06.08.2007	F	41	S1
51464	<20	588	3TC,ETV,RAL	76	1	0	249000	23.101996	M	69	S2
52353	<20	843	TDF,FTC,RAL	41	0	0	136000	05.02.2010	M	56	S3
52227	<20	1369	ABC,3TC,ETV	61	0	0	3760000	15.05.2008	M	41	S4
50080	<20	324	TDF,FTC,DRV,RTV,ETV	75	3	0	159000	01.06.1986	M	56	P1
52410	30	644	TDF,FTC,EFV	21	1	0	47900	18.03.2011	M	42	P1
52447	<20	507	TDF,FTC,DRV,RTV,MVC	24	2	0	582000	01.07.2003	F	37	P2
51736	<20	463	TDF,FTC,ATV,RTV	86	2	2	312000	15.10.1995	M	59	P2
52284	<20	595	TDF,FTC,RAL	55	0	0	121000	11.03.2009	M	43	P2
52378	<20	834	TDF,FTC,ATV,RTV	35	0	0	5250000	02.08.2010	M	34	P2
51555	<20	667	ABC,3TC,DRV,RTV,MVC	39	6	1	32700	07.10.1997	M	58	P3
52478	<20	761	TDF,FTC,ATV,RTV	13	0	0	230000	15.05.2009	M	43	P3
52160	<20	1193	ABC,3TC,ETV	62	3	1	93700	25.10.2006	M	45	S5
52365	<20	650	TDF,FTC,EFV	36	2	0	123000	08.02.2006	M	41	P4
52368	<20	853	ABC,3TC,ETV	50	0	1	196000	15.03.2003	M	46	P4
51554	<20	942	TDF,FTC,DRV,RTV,ETV	72	0	1	74000	09.09.1997	M	40	S6
51579	<20	925	TDF,FTC,NVP	173	2	5	504878	26.01.1998	F	47	P5
51612	<20	804	TDF,FTC,EFV	21	0	0	68800	15.12.1997	M	42	P5
52241	<20	240	ABC,3TC,DRV,RTV	55	0	0	140000	20.05.2008	F	36	P5
