# Supplementary material for: Dynamics of HIV Latency and Reactivation in a Primary CD4+ T Cell Model
Source: PLoS Pathog. 2014 May 29;10(5):e1004156. doi: 10.1371/journal.ppat.1004156 (PMC4038609; doi:10.1371/journal.ppat.1004156)
Supplement: Figure S3 — Features of HIV transcription under several reactivation agents. Panel A. Distribution of HIV reads along the vector genome; each panel compares one agent against DMSO as control. On the top is depicted the viral vector genome used. TSS: transcription start site; D: splice donor; A: splice acceptor. Reads mapping to the LTR are equally assigned to 5′ and 3′ ends. Panel B. Pattern of splicing for the main viral RNA forms: genomic unspliced full-length viral RNA (US, blue), singly spliced RNAs without the Gag-Pol major intron (SS, green; spliced in D1 but not in D4), and multiply spliced subgenomic mRNAs (MS, red; spliced in D1 and in D4). (PDF) [file ppat.1004156.s003.pdf]

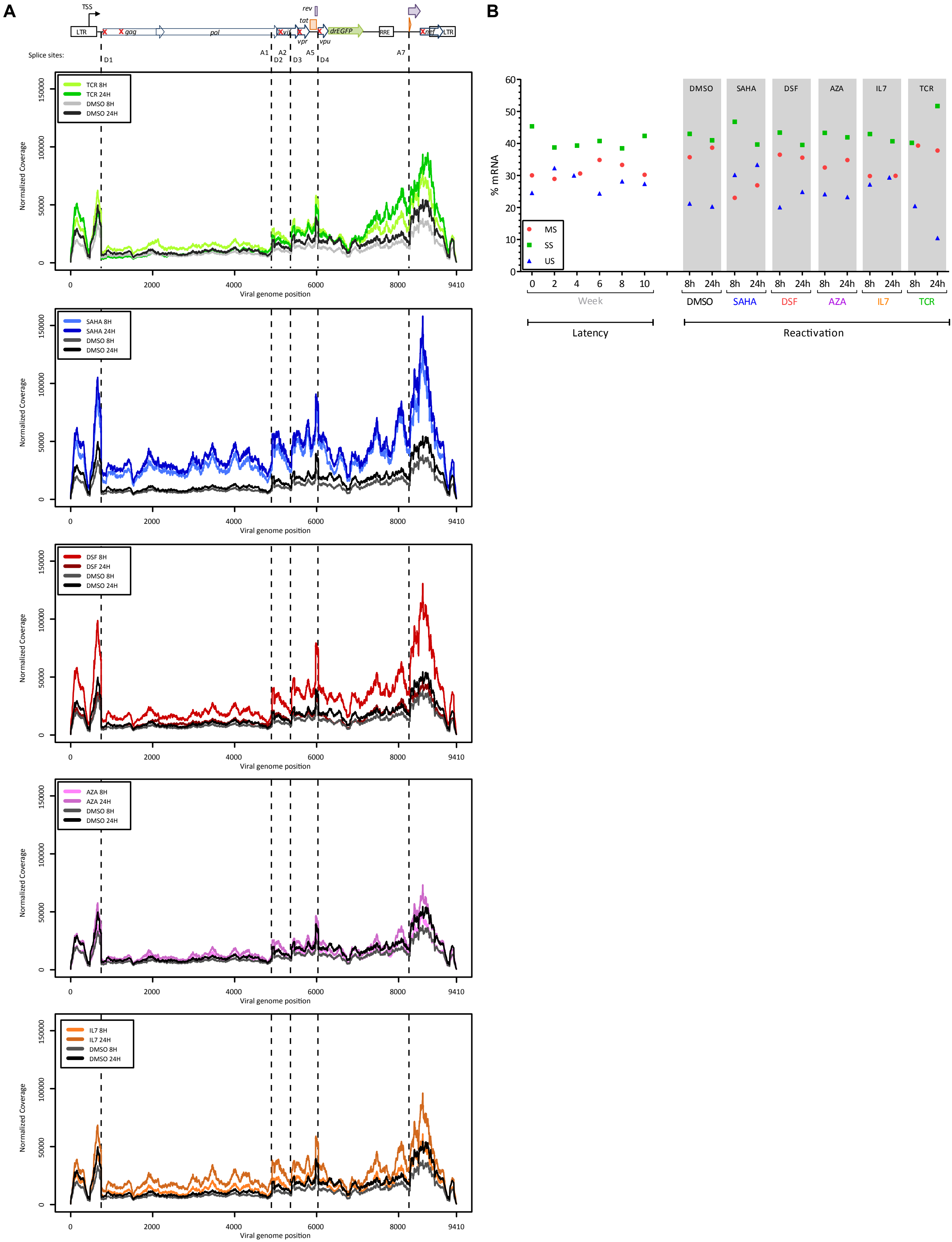
A
TSS
LTR
gag
pol
vif
vpr
tat
rev
vpu
drEGFP
RRE
nef
LTR
Splice sites:
D1
A1
D2
A2
D3
A5
D4
A7
TCR 8H
TCR 24H
DMSO 8H
DMSO 24H
SAHA 8H
SAHA 24H
DSF 8H
DSF 24H
AZA 8H
AZA 24H
IL7 8H
IL7 24H
Normalized Coverage
Viral genome position
0
2000
4000
6000
8000
9410
0
50000
100000
150000
B
% mRNA
MS
SS
US
DMSO
SAHA
DSF
AZA
IL7
TCR
0
2
4
6
8
10
Week
8h
24h
Latency
Reactivation
